# Phosphorylation of the Anaphase Promoting Complex activator FZR1/CDH1 is required for Meiosis II entry in mouse male germ cell

**DOI:** 10.1038/s41598-020-67116-0

**Published:** 2020-06-22

**Authors:** Nobuhiro Tanno, Shinji Kuninaka, Sayoko Fujimura, Kazumasa Takemoto, Kaho Okamura, Naoki Takeda, Kimi Araki, Masatake Araki, Hideyuki Saya, Kei-ichiro Ishiguro

**Affiliations:** 1 Department of Chromosome Biology, Institute of Molecular Embryology and Genetics (IMEG), Kumamoto University, Kumamoto, 860-0811 Japan; 2 Division of Gene Regulation, Institute for Advanced Medical Research, Keio University School of Medicine, 160-8582 Tokyo, Japan; 3 Liaison Laboratory Research Promotion Center, IMEG, Kumamoto University, Kumamoto, 860-0811, Japan; 4 Institute of Resource Development and Analysis, Kumamoto University, Kumamoto, 860-0811 Japan; 5 Center for Metabolic Regulation of Healthy Aging, Kumamoto University, Kumamoto, 860-0811 Japan

**Keywords:** Cell division, Meiosis, Germline development, Spermatogenesis

## Abstract

FZR1/CDH1 is an activator of Anaphase promoting complex/Cyclosome (APC/C), best known for its role as E3 ubiquitin ligase that drives the cell cycle. APC/C activity is regulated by CDK-mediated phosphorylation of FZR1 during mitotic cell cycle. Although the critical role of FZR1 phosphorylation has been shown mainly in yeast and *in vitro* cell culture studies, its biological significance in mammalian tissues *in vivo* remained elusive. Here, we examined the *in vivo* role of FZR1 phosphorylation using a mouse model, in which non-phosphorylatable substitutions were introduced in the putative CDK-phosphorylation sites of FZR1. Although ablation of FZR1 phosphorylation did not show substantial consequences in mouse somatic tissues, it led to severe testicular defects resulting in male infertility. In the absence of FZR1 phosphorylation, male juvenile germ cells entered meiosis normally but failed to enter meiosis II or form differentiated spermatids. In aged testis, male mutant germ cells were overall abolished, showing Sertoli cell-only phenotype. In contrast, female mutants showed apparently normal progression of meiosis. The present study demonstrated that phosphorylation of FZR1 is required for temporal regulation of APC/C activity at meiosis II entry, and for maintenance of spermatogonia, which raised an insight into the sexual dimorphism of FZR1-regulation in germ cells.

## Introduction

Anaphase promoting complex/Cyclosome (APC/C) controls timely transitions of mitotic cell cycle phases by promoting ubiquitylation and degradation of many key cell cycle regulators^1^. APC/C activity is regulated by either of two co-activators CDC20 and FZR1(CDH1), which determine the substrate specificity of ubiquitylation for each cell cycle phase^2–5^. APC/C^CDC20^ activity appears in metaphase-to-anaphase transition, when it has an essential function in promoting chromosome segregation by mediating cyclin B1 and securin degradation. APC/C ^FZR1^ is thought to regulate a wide range of cell cycle events, in which significant number of proteins have been identified as APC/C^FZR1^ substrates^6,7^.

While CDC20 plays a role in APC/C activity at metaphase, when the cyclin-dependent kinase 1 (CDK1) activity is high, FZR1 contributes to APC/C activity when CDK1 activity is sustained at a low level^4,8^. APC/C^FZR1^ activity is negatively regulated by CDK-mediated phosphorylation of FZR1 during mitotic cell cycle. At mitotic exit, reduction of CDK1 activity leads to phosphatase-mediated dephosphorylation of FZR1 which then binds and activates APC/C until late G1 phase^9^. In yeast mitotic cell cycle, FZR1 is phosphorylated by increased level of CDK activity after late G1/S onward, which subsequently leads to dissociation of FZR1 from APC/C and its inactivation^10–15^. In mammals, mitotic cells transiently transfected with mutant FZR1 (CDH1) that lacked potential CDK1 phosphorylation sites, resulted in premature reduction of cyclin A and cyclin B with decrease in G2/M phase-cells^16,17^. Thus, CDK-mediated phosphorylation of FZR1 plays a crucial role in temporal regulation of APC/C activity during mitotic cell cycle. However, the physiological significance of FZR1 phosphorylation is yet to be examined in vertebrate mitotic tissues *in vivo*.

The meiotic cell cycle consists of one round of DNA replication followed by two rounds of chromosome segregation, producing haploid gametes from diploid cells. Although study in budding yeast elucidated that the role of APC/C in meiosis relies on the meiosis-specific co-activator AMA1^18,19^, its homolog or counterpart does not exist in mammals. Instead, CDC20 and FZR1 contribute to the regulation of APC/C activity in mammalian meiosis. In mice, it was demonstrated that loss of FZR1 led to abnormal spermatogonial proliferation and defects in progression of meiotic prophase I in both male and female germ cells^20^. In female meiosis I, FZR1 plays crucial roles in sustaining dictyate arrest of germinal vesicle (GV) oocytes^21–23^ and in regulating chromosome segregation^24–28^. These studies indicate that FZR1 is required for mammalian meiosis, but it remains elusive how APC/C activity regulated by phosphorylation of FZR1 is involved in meiotic cell cycle.

Here, we examined whether phosphorylation of FZR1 is required for the regulation of APC/C activity during mitotic and meiotic cell cycle *in vivo*, using knockin mice that carries non-phosphorylatable mutations of FZR1. Our study demonstrates that phosphorylation of FZR1 is required for maintenance of spermatogonia for a long period of time. Furthermore, we show that FZR1 phosphorylation has a crucial role in the regulation of APC/C activity during meiosis I-meiosis II transition in juvenile male but not in female. Sexual dimorphism in the requirement of FZR1 phosphorylation raised an insight into different modes of meiosis I-to-meiosis II progression in spermatocytes and oocytes.

## Results

### Generation of *Fzr1*^9A/9A^ knockin mice

**Figure 1:**
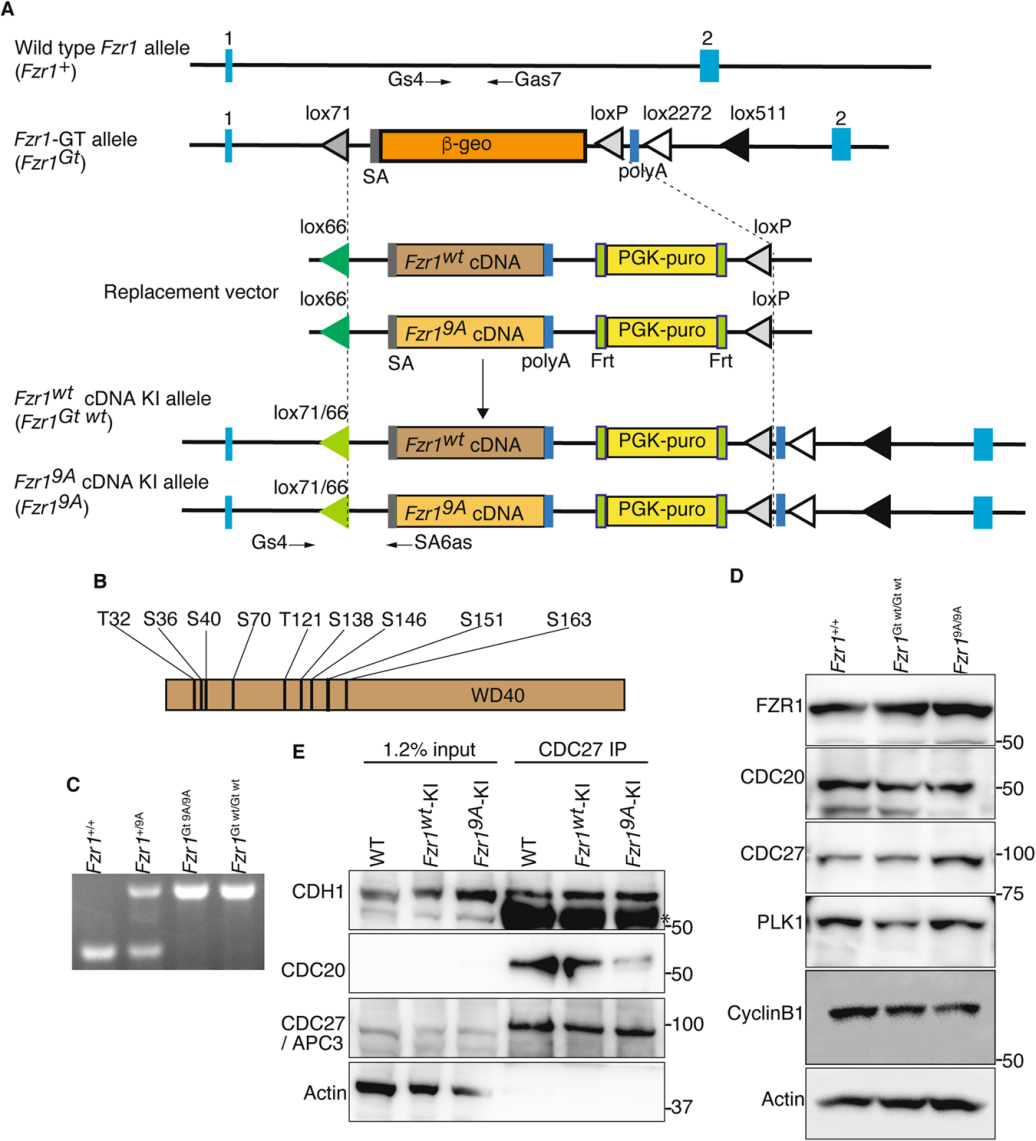
Generation of *Fzr1*^9A/9A^ knockin mice. **(A)**Schematic representation of the WT *Fzr1*^+^ allele, the Gene trapped (GT) allele *Fzr1*^GT^, the knock-in (KI) alleles of *Fzr1*^GT wt^ and *Fzr1*^9A^ and the replacement vectors possessing *Fzr1*^GT wt^ or *Fzr1*^9A^ cDNA. *Fzr1* ^GT^ allele was generated by integration of the exchangeable Gene Trap (GT) vector in the 1st intron of the endogenous *Fzr1* locus^30^. Cre-mediated recombination between *Fzr1*^GT^ allele and the replacement vector generated *Fzr1*^9A^ KI allele. The control *Fzr1*^wt^ allele was generated between *Fzr1*^GT^ allele and the replacement vector possessing *Fzr1* ^wt^ cDNA in the same manner. SA: splicing acceptor, Frt: Flippase recombination site, polyA: poly adenylation signal, *lox* P and its variant recombination sites are indicated by triangles. Light blue rectangles indicate the exons of *Fzr1* locus. Arrows indicate the PCR primers for genotyping. (**B**) Nine putative CDK-phosphorylated sites of Ser and Thr residues in FZR1, where Ala substitutions were introduced. (**C**) PCR genotyping of genomic DNA from *Fzr1*^+/+^, *Fzr1*^+/9A^, *Fzr1*^9A/9A^ and *Fzr1*^Gt wt/Gt wt^ KI mice. **(D)** Western blot of testis extracts from *Fzr1*^+/+^, *Fzr1*^Gt wt/Gt wt^ and *Fzr1*^9A/9A^ testes (P18), probed by antibodies as indicated. **(E)** Western blot of immunoprecipitates from testis extracts of *Fzr1*^+/+^, *Fzr1*^Gt wt/Gt wt^ and *Fzr1*^9A/9A^ mice using anti-CDC27 antibody. *Indicates nonspecific bands cross-reacted with IgG. See also Supplementary Fig. S2 for the uncropped images.

It has been shown that human FZR1 is phosphorylated during mitotic cell cycle. Ectopic expression of non-phosphorylatable FZR1 mutant in HeLa cells resulted in formation of constitutively active APC/C^FZR1^ and a reduction of G2/M phase^17^. To analyze the physiological role of FZR1-phosphorylation, we generated a mouse model, *Fzr1*^9A/9A^ knockin (*Fzr1*^9A/9A^ KI) mice, in which the nine putative CDK-phosphorylation sites of Ser/Thr residues in F*zr*1 were substituted with alanine amino acids (Fig. 1A–C). *Fzr1*^9A/9A^ KI allele was generated by Cre-mediated site-specific recombination of full length cDNA encoding mutant *Fzr1*^9A^ into the endogenous *Fzr1* locus using an exchangeable gene-trap (GT) line^29,30^. For the control mouse line, full length cDNA cassette encoding wild type (WT) FZR1 was inserted into the targeted locus in the same manner, generating *Fzr1*^Gt wt/Gt wt^ KI mice (Fig. 1A). To distinguish *Fzr1*^Gt wt^ KI allele from natural WT *Fzr1* allele, hereafter we refer to natural WT *Fzr1* allele as *Fzr1*^+^. The expression levels of FZR1-Gt wt and FZR1–9A proteins in the corresponding KI testes were comparable to the FZR1 expression level in natural WT *Fzr1*^+/+^ testes at postnatal day18 (P18) (Fig. 1D). Further, CDC20, APC/C subunits (CDC27/APC3) and the canonical substrates of APC/C (Cyclin B1, PLK1) overall showed similar expression levels in natural WT *Fzr1*^+/+^, *Fzr1*^9A/9A^ KI and *Fzr1* ^Gt wt/Gt wt^ KI testes (Fig. 1D). We also confirmed *Fzr1*^Gt wt/Gt wt^ KI mice showed normal fertility as natural WT *Fzr1*^+/+^, indicating *Fzr1*^Gt wt^ KI allele functions in a manner indistinguishable from natural WT *Fzr1*^+^ allele. Immunoprecipitation of CDC27/APC3, a core subunit of APC/C, demonstrated that the non-phoshorylatable FZR1–9A and control FZR1-Gt wt proteins expressed from the corresponding KI alleles were indeed incorporated in APC/C of *Fzr1*^9A/9A^ and *Fzr1*^Gt wt/Gt wt^ KI testes, respectively (Fig. 1E). We noticed that the level of CDC20 incorporated in APC/C in *Fzr1*^9A/9A^ testis was less than that in natural WT *Fzr1*^+/+^ and *Fzr1*^Gt wt/Gt wt^ KI testes. This implies that persistent inclusion of FZR1–9A in APC/C impedes the association of CDC20 to APC/C.

### Introduction of non-phosphorylatable mutations of FZR1 leads to male infertility

Inter-crossing of heterozygous (*Fzr1*^9A/+^) males and females produced average 7.285 litter size with normal number of litters of *Fzr1*^+/+^, *Fzr1*^+/9A^ and *Fzr1*^9A/9A^ genotypes expected by mendelian ratio (total *Fzr1*^+/+^:11 pups, *Fzr1*^+/9A^: 28 pups and *Fzr1*^9A/9A^: 12 pups from 7 pairs of intercrossing. *p* = 0.9557 by chi-square test). The homozygous *Fzr1*^9A/9A^ KI mice developed normally without any overt phenotype in adult somatic tissues, suggesting that FZR1–9A showed little impact on mouse development and mitotic cell cycle of mouse somatic tissues.

**Figure 2:**
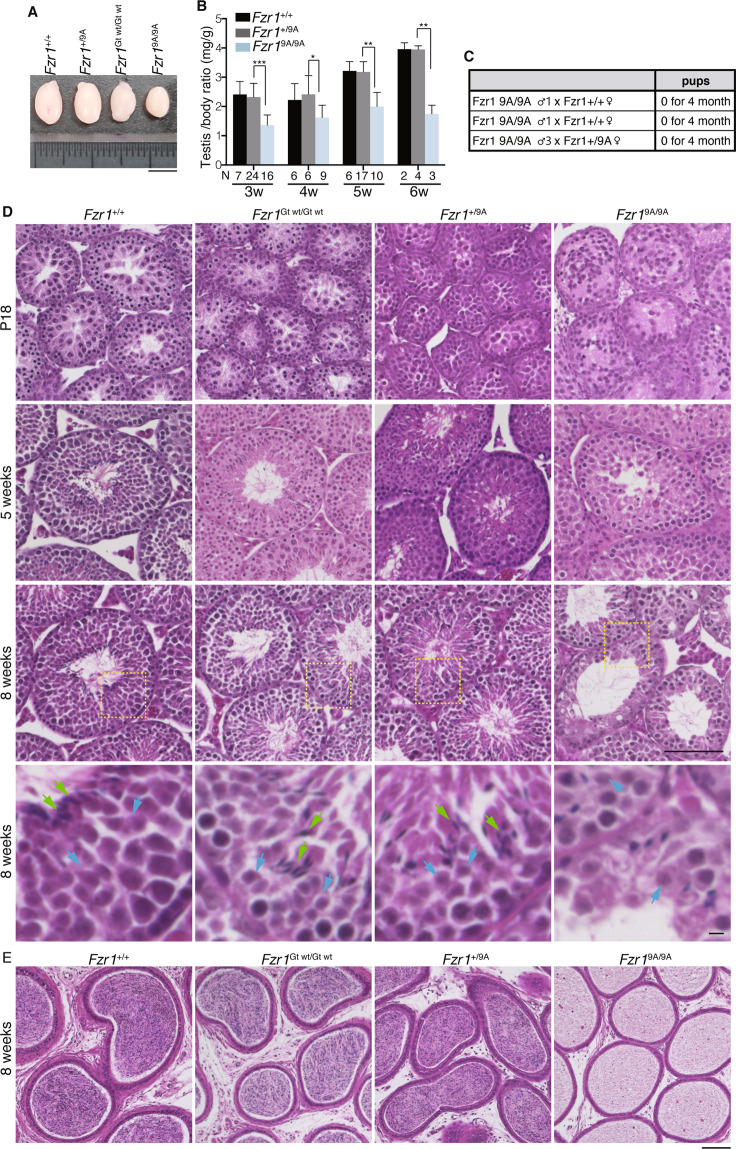
Spermatogenesis is defective in *Fzr1*^9A/9A^ knockin mice. **(A)** Gross morphology of representative testes from 8-week-old control *Fzr1*^+/+^, *Fzr1*^+/9A^, *Fzr1*^Gt wt/Gt wt^ and *Fzr1*^9A/9A^ KI mice. Scale bar, 5 mm. **(B)** Testis /body weight ratio (mg/g) of control *Fzr1*^+/+^, *Fzr1*^+/9A^ and *Fzr1*^9A/9A^ KI testes at the indicated ages. N: number of animals examined. *p*-values are shown (paired t-test). ***p < 0.001, **p < 0.01, *p < 0.05. **(C)** Number of pups born by mating *Fzr1*^9A/9A^ KI males with wild type or *Fzr1*^9A/+^ females to examine fertility. **(D)** Hematoxylin and Eosin stained histological sections of seminiferous tubules in WT, *Fzr1*^Gt wt/Gt wt^, *Fzr1*^+/9A^ and *Fzr1*^9A/9A^ KI mice at the indicated ages. Scale bar: 100 μm. Enlarged images of the area indicated by yellow dot line are shown on the bottom with scale bar: 5 μm, Green arrow: spermatozoa, Blue arrow: round spermatid. **(E)** Hematoxylin and Eosin stained sections of the epididymis in WT, *Fzr1*^Gt wt/Gt wt^, *Fzr1*^+/9A^ and *Fzr1*^9A/9A^ KI mice at the age of 8 weeks. Scale bar: 100 μm.

Notably, although testes in juvenile *Fzr1*^9A/9A^ KI males did not show apparent difference from those of control WT *Fzr1*^+/+^ or *Fzr1*^+/9A^, defects in male reproductive organs were evident with smaller-than-normal testes at adulthood (Fig. 2A). Remarkably, testes in the *Fzr1*^9A/9A^ KI males at 3 week or older were significantly smaller compared to the age-matched *Fzr1*^+/+^ males (Fig. 2B). Since it is known that the first wave of spermatogenesis completes meiotic prophase and reaches to meiotic divisions generating haploid spermatids at earliest P18, the observation in *Fzr1*^9A/9A^ KI males suggests that the primary defect of *Fzr1*^9A/9A^ KI males emerges during or immediately after meiotic prophase.

To determine whether the introduction of non-phosphorylatable mutations in FZR1 affected male fertility, 8-week-old *Fzr1*^9A/9A^ KI males, control WT *Fzr1*^+/+^ males and *Fzr1*^Gt wt/Gt wt^ KI males were mated with C57BL6 wild-type or corresponding control females over a period of 4 months. While the control WT *Fzr1*^+/+^ and *Fzr1*^Gt wt/Gt wt^ KI males produced normal size of litters over this period, the *Fzr1*^9A/9A^ KI males showed infertility over a period of 4 months (Fig. 2C). Since the control *Fzr1*^Gt wt/Gt wt^ KI mice showed normal fertility, it is tenable that the infertility in the *Fzr1*^9A/9A^ KI males was attributed to the introduction of non-phosphorylatable mutations in FZR1. Since heterozygous *Fzr1*^9A/+^ KI males showed normal fertility, the phenotype exhibited by the *Fzr1*^9A^ KI allele was recessive rather than dominant negative in terms of male fertility (Fig. 2B).

Histological analysis revealed that obvious difference was not observed between *Fzr1*^9A/9A^ KI and the control seminiferous tubules at P18 (Fig. 2D), suggesting that the first wave of spermatogenesis normally progressed through meiotic prophase in *Fzr1*^9A/9A^ KI testis. However, at 5 week-old onward, spermatozoa were absent in the *Fzr1*^9A/9A^ KI seminiferous tubules, whereas spermatogonia, spermatocytes and round spermatid-like cells appeared. This observation was in sharp contrast to those of the age-matched natural WT *Fzr1*^+/+^ and the control *Fzr1*^Gt wt/Gt wt^ KI males (Fig. 2D). Thus, spermatogenesis was severely impaired in the *Fzr1*^9A/9A^ KI seminiferous tubules. Accordingly, spermatozoa were completely absent in *Fzr1*^9A/9A^ KI epididymis (Fig. 2E), corroborating the infertility of the *Fzr1*^9A/9A^ KI males. These observations suggested that although the germ cells had undergone spermatogonial differentiation and meiosis, they failed to progress spermiogenesis beyond round spermatids in the *Fzr1*^9A/9A^ KI seminiferous tubules at younger age. Therefore, we reasoned that spermatogenesis defects in *Fzr1*^9A/9A^ KI male could be attributed to the failure of differentiation from spermatocyte to round spermatids or in later stage during the juvenile age. Altogether, phosphorylation of FZR1 is required for normal progression of spermatogenesis.

In contrast to the male phenotype, *Fzr1*^9A/9A^ KI females were fertile when mated with heterozygous male, and showed apparently no overt histological defects in adult ovaries (Supplementary Fig. S1A,B), although we could not exclude the possibility that more subtle defects might have occurred in the ovaries besides fertility. Thus the infertility caused by non-phosphorylatable mutations in FZR1 was male specific.

### Phosphorylation of FZR1 is required for meiosis I-meiosis II transition in male

**Figure 3:**
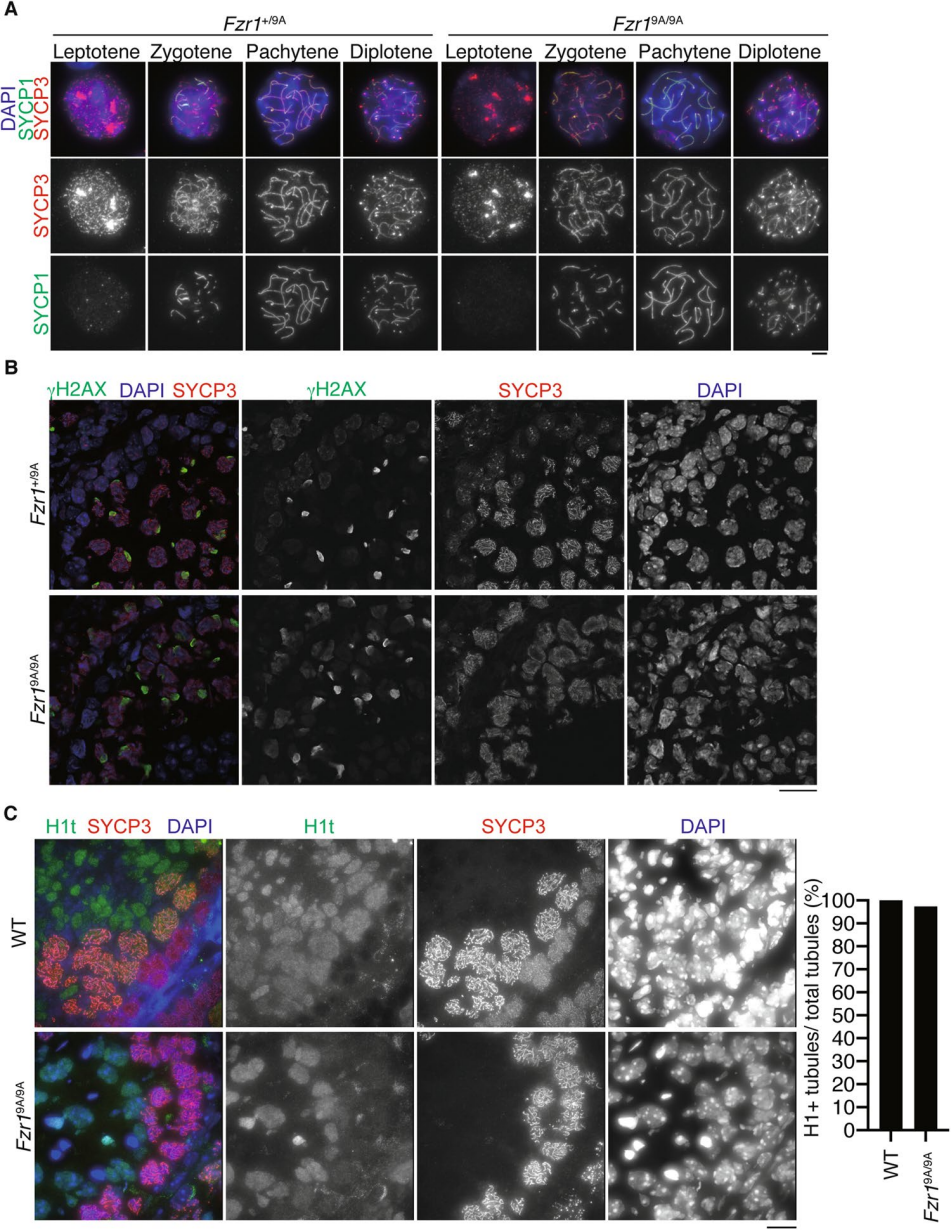
Normal progression of meiotic prophase in *Fzr1*^9A/9A^ KI males. Spread nuclei from the testes of *Fzr1*^+/9A^ and *Fzr1*^9A/9A^ KI mice (P28) were immunostained for SYCP3, SYCP1 and DAPI. Scale bar: 5 μm. (**A**) Seminiferous tubule sections of *Fzr1*^+/9A^ and *Fzr1*^9A/9A^ KI mice (3-week-old) were immunostained for SYCP3, γH2AX and DAPI. Scale bar: 20 μm. (**B**) Seminiferous tubule sections in WT and *Fzr1*^9A/9A^ KI mice (5-week-old) were immunostained for SYCP3, Histone H1t, SYCP1 and DAPI. Quantification of the seminiferous tubules that have H1t +/SYCP3 + cells per the seminiferous tubules that have SYCP3 + spermatocyte cells in WT (n = 1) and *Fzr1*^9A/9A^ KI mice (n = 1) testes (bar graph). Scale bar: 15 *μ*m.

**Figure 4:**
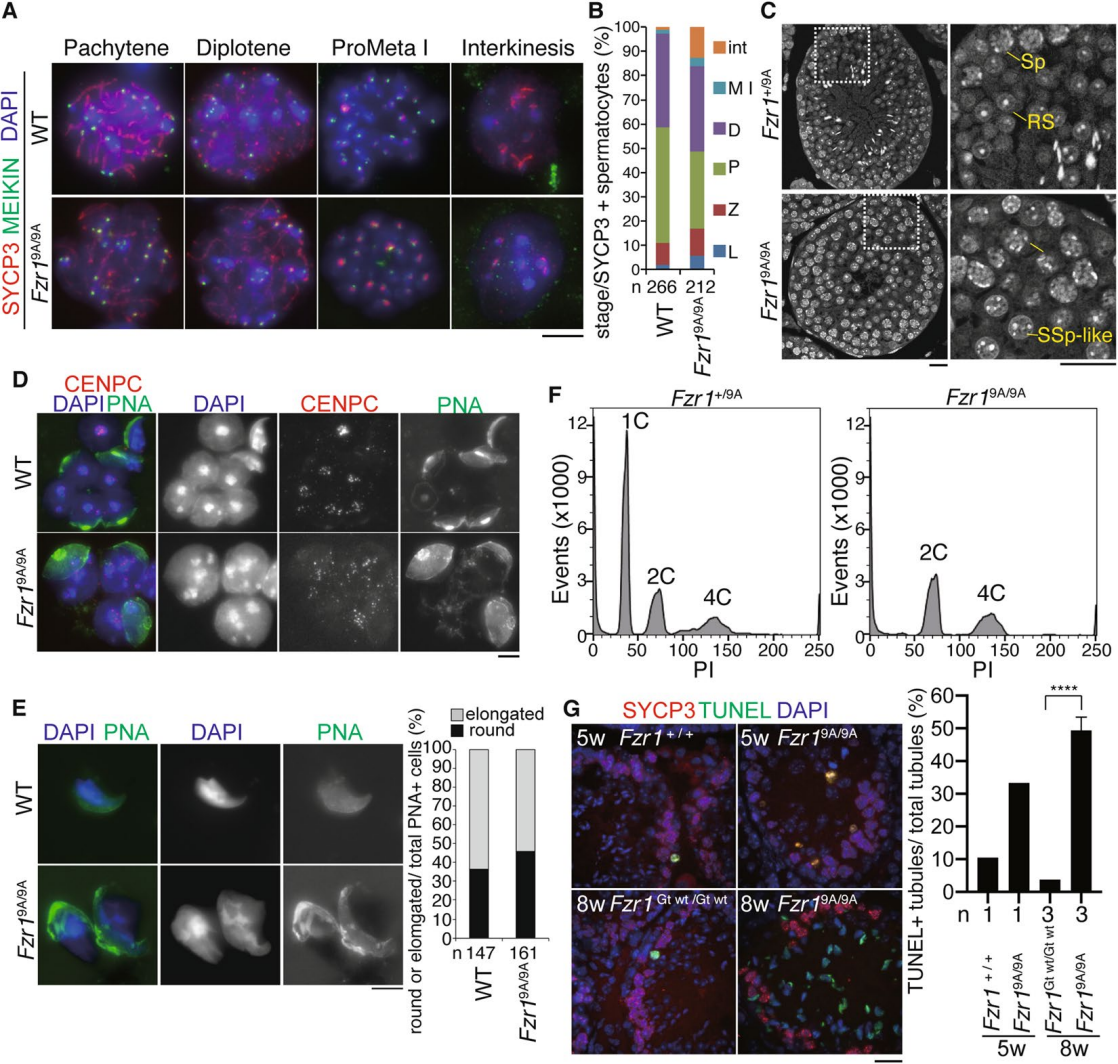
*Fzr1*^9A/9A^ knockin testes show defect in meiosis I-meiosis II transition. (**A**)Spread nuclei from the testes of wild type and *Fzr1*^9A/9A^ KI mice (10-week-old) were immunostained for SYCP3, MEIKIN, and DAPI. Scale bar: 5 μm. (**B**) Quantification of developmental stage per total SYCP3 + spermatocytes of wild type and *Fzr1*^9A/9A^ KI mice (8-week-old) is shown. n indicates the number of examined cells. L: leptotene, Z: zygotene, P: pachyetene, D: diplotene, M I: Metaphase I, int: interkinesis. (**C**) Seminiferous tubule sections in *Fzr1*^+/9A^ and *Fzr1*^9A/9A^ KI mice (6-week-old) were DAPI-stained. Enlarged images are shown on the right to highlight the secondary-spermatocyte like cells in *Fzr1*^9A/9A^ KI mice. Sp: spermatocyte, RS: round spermatid, SSp-like: secondary spermatocyte-like cell. Scale bar: 20 μm. (**D**) The round spermatid and the secondary spermatocyte-like cells from the testes of WT and *Fzr1*^9A/9A^ KI mice (14-week-old), respectively, were spread as in (A) and immunostained for Centromere protein-C (CENPC), PNA lectin and DAPI. Scale bar: 5 μm. (**E**) The elongated spermatid and the elongated spermatid-like cells from the testes of WT and *Fzr1*^9A/9A^ KI mice (8-week-old), respectively, were immunostained for PNA lectin and DAPI. Quantification of round spermatid (-like cells) or elongated spermatid (-like cells) per total PNA+ cells is shown. n indicates the number of examined cells. Scale bar: 5 μm. (**F**) Flow cytometry histogram of propidium-iodide-stained testicular cells isolated from *Fzr1*^+/9A^ and *Fzr1*^9A/9A^ KI mice (P28). C indicates DNA content in the testicular cell population. (**G**) Seminiferous tubule sections from control *Fzr1*^+/+^ (5w), control *Fzr1* ^Gt wt/Gt wt^ (8w) and *Fzr1*^9A/9A^ KI testes (5w, 8w) were stained for SYCP3, TUNEL and DAPI. Quantification of the seminiferous tubules that have TUNEL + cells per total tubules.n indicates the number of examined animals. ****p < 0.0001 (t-test). Scale bar: 25 μm.

Given that spermatogenesis defects in *Fzr1*^9A/9A^ KI male might arise during or after meiosis in the juvenile age, we further investigated at which stage the primary defect appeared in juvenile *Fzr1*^9A/9A^ KI. We analyzed the progression of spermatogenesis in juvenile *Fzr1*^9A/9A^ KI males by immunostaining using stage specific markers. Consistent with above observations, immunostaining analysis with antibodies against SYCP3 (a component of meiotic chromosome axis) along with γH2AX (a marker of double strand break for meiotic recombination) or SYCP1 (a marker of homologous chromosome synapsis) demonstrated that spermatocytes underwent meiotic recombination and homologous chromosome synapsis in juvenile *Fzr1*^9A/9A^ KI males, which were comparable to age-matched control (Fig. 3A,B). The *Fzr1*^9A/9A^ KI spermatocytes reached mid to late pachytene stage as indicated by testis-specific histone H1t, which were also comparable to age-matched WT (Fig. 3C). Thus, spermatocytes progressed the meiotic prophase normally in juvenile *Fzr1*^9A/9A^ KI males. We further investigated the meiotic processes before and after the first meiotic division. Spread nuclei from the testes were immunostained with antibodies against SYCP3 along with MEIKIN, a kinetochore marker of late pachytene-metaphase I^31^. In *Fzr1*^9A/9A^ KI, spermatogenesis reached metaphase I (centromeric SYCP3 + /MEIKIN + ) and interkinesis (bar-like SYCP3 + /MEIKIN-)(Fig. 4A), an interphase-like stage between meiosis I and meiosis II^32^, suggesting that at least meiosis I was complete in *Fzr1*^9A/9A^ KI spermatocytes. Notably, among the SYCP3 + spermatocytes, interkinesis spermatocytes were accumulated in *Fzr1*^9A/9A^ KI compared to WT (*p* < 0.0001, chi-square test) (Fig. 4B). Intriguingly, although *Fzr1*^9A/9A^ KI spermatocytes reached interkinesis at the most advanced stage, we noticed that atypical secondary spermatocyte-like cells appeared in the *Fzr1*^9A/9A^ KI (Fig. 4C). Whereas WT round spermatids mostly exhibited single chromocenters (the clusters of centromeric heterochromatin), *Fzr1*^9A/9A^ KI secondary spermatocyte-like cells showed multiple number of chromocenters and exhibited round spermatid-like morphology (Fig. 4D). We also observed a small fraction of elongated spermatid-like cells with aberrant shape in *Fzr1*^9A/9A^ KI testis (Fig. 4E). Notably, since *Fzr1*^9A/9A^ KI secondary spermatocyte-like cells and spermatid-like cells were PNA lectin (a marker of spermatid) positive (Fig. 4D,E), spermiogenesis program at least in part progressed in *Fzr1*^9A/9A^ KI atypical spermatid-like cells. Crucially, flow cytometry analysis of testicular cells from *Fzr1*^9A/9A^ KI testes (d28) demonstrated complete absence of the 1 C population, despite the existence of spermatid-like cells (Fig. 4F). Therefore, we reasoned that the observed primary defect in *Fzr1*^9A/9A^ KI spermatogenesis derived from a process during or at the entry into meiosis II rather than a failure in post-meiotic differentiation into elongated spermatid. Because we did not observe any population with 1 C DNA content in *Fzr1*^9A/9A^ KI, secondary spermatocyte-like cells or spermatid-like cells were produced after normal meiosis I chromosome segregation exhibiting 2 C DNA contents. It is plausible that secondary spermatocytes failed to progress from the interkinesis stage to meiosis II, although spermatid program might have been turned on in *Fzr1*^9A/9A^ KI testes. Furthermore, a subpopulation of those secondary spermatocyte-like cells was TUNEL positive in *Fzr1*^9A/9A^ KI seminiferous tubules, suggesting that those aberrant secondary spermatocyte-like cells and spermatid-like cells were eliminated at least in part by apoptosis (Fig. 4G).

### Phosphorylation of FZR1 is required for the entry into meiosis II in male meiosis

**Figure 5:**
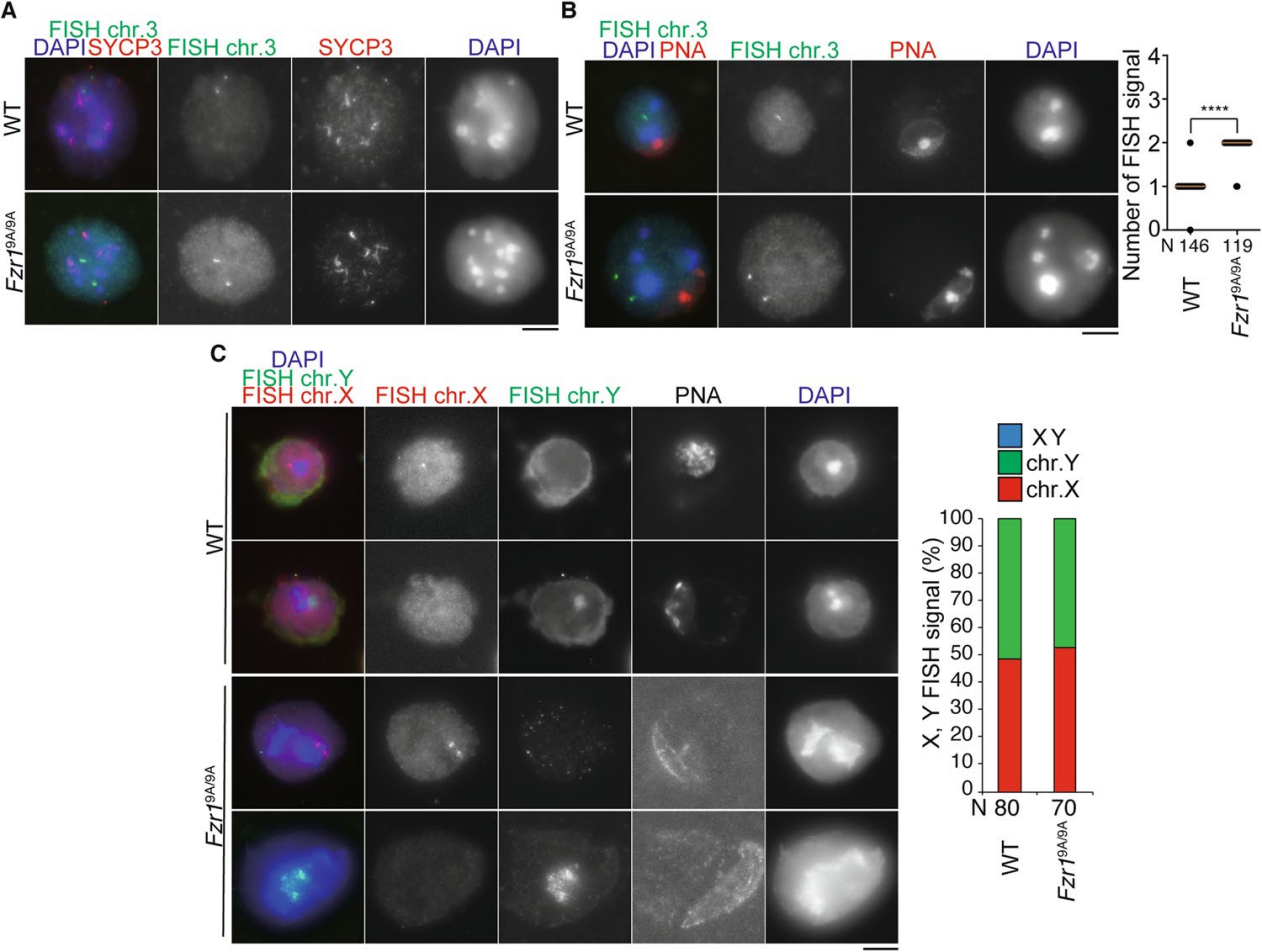
*Fzr1*^9A/9A^ KI spermatocytes lead to sister chromatid non-disjunction. **(A)** Interkinesis spermatocytes from WT (*Fzr1*^+/+^) and *Fzr1*^9A/9A^ KI (14-week old) immunostained with SYCP3 (red) were subjected to FISH with a point probe (Chr.3) (green). **(B)** WT (*Fzr1*^+/+^) round spermatids and *Fzr1*^9A/9A^ KI secondary-spermatocyte-like cells (14-week old) immunostained with SYCP3 and PNA were subjected to FISH with a point probe (Chr.3) (green). The number of FISH signals in WT round spermatids and *Fzr1*^9A/9A^ KI secondary-spermatocyte-like cells are represented in a scatter plot with medians. N: number of examined cells. ****p < 0.0001 (t-test). **(C)** WT (*Fzr1*^+/+^) round spermatids and *Fzr1*^9A/9A^ KI (8-week old) secondary-spermatocyte-like cells stained with PNA were subjected to FISH with a point probe that detects X chromosome (red) and a whole painting probe that detects Y chromosome (green). The number of the cells that exhibited FISH signal of X only, Y only or both are quantified in a bar graph. *p* = 0.569 (chi-square test). N indicates the number of examined cells. Scale bars: 5 μm.

Given that *Fzr1*^9A/9A^ KI spermatocytes undergo meiosis I but do not produce 1 C DNA content-spermatids, the secondary spermatocyte-like cells may be a consequence of the failure in meiosis II. Homologous chromosomes are segregated in meiosis I, whereas sister chromatids are separated in meiosis II. In order to examine the chromosome composition in *Fzr1*^9A/9A^ KI secondary spermatocyte-like cells, we performed immuno-FISH assays using a probe that detects a specific DNA sequence in the mid-arm region of chromosomes 3. In both WT and *Fzr1*^9A/9A^ KI mice, interkinesis spermatocytes showed a pair of FISH signals (Fig. 5A), indicating that homologous chromosomes or sister chromatids were disjoined after meiosis I. In contrast, while WT round spermatids showed a single FISH signal, most of *Fzr1*^9A/9A^ KI secondary spermatocyte-like cells showed a pair of FISH signals (Fig. 5B), suggesting that in *Fzr1*^9A/9A^ KI interkinesis spermatocytes failed to enter meiosis II. Moreover, immuno-FISH assays using probes that detect a centromere proximal region of X chromosome and whole Y chromosome showed that *Fzr1*^9A/9A^ KI secondary spermatocyte-like cells have either X or Y chromosome, but not both (Fig. 5C). This suggests that homologous chromosomes rather than sister chromatids were disjoined in meiosis I in *Fzr1*^9A/9A^ KI as normal spermatocytes. Thus, in *Fzr1*^9A/9A^ KI male, homologous chromosomes underwent disjunction at meiosis I, but failure in meiosis II entry consequently led to non-disjunction of sister chromatids. These results suggested that phosphorylation of FZR1 is required for the progression from interkinesis to meiosis II in male meiosis. In mitotic cell cycle, constitutively active APC/C^FZR1^ reduces cyclin A and cyclin B levels prematurely with decrease in G2/M phase cells^17^. Our observations in *Fzr1*^9A/9A^ KI male implies that without phosphorylation of FZR1, APC/C^FZR1^ substrates might undergo premature degradation, which impairs the entry into meiosis II in male.

### Phosphorylation of FZR1 is required for maintaining spermatogonia population

**Figure 6:**
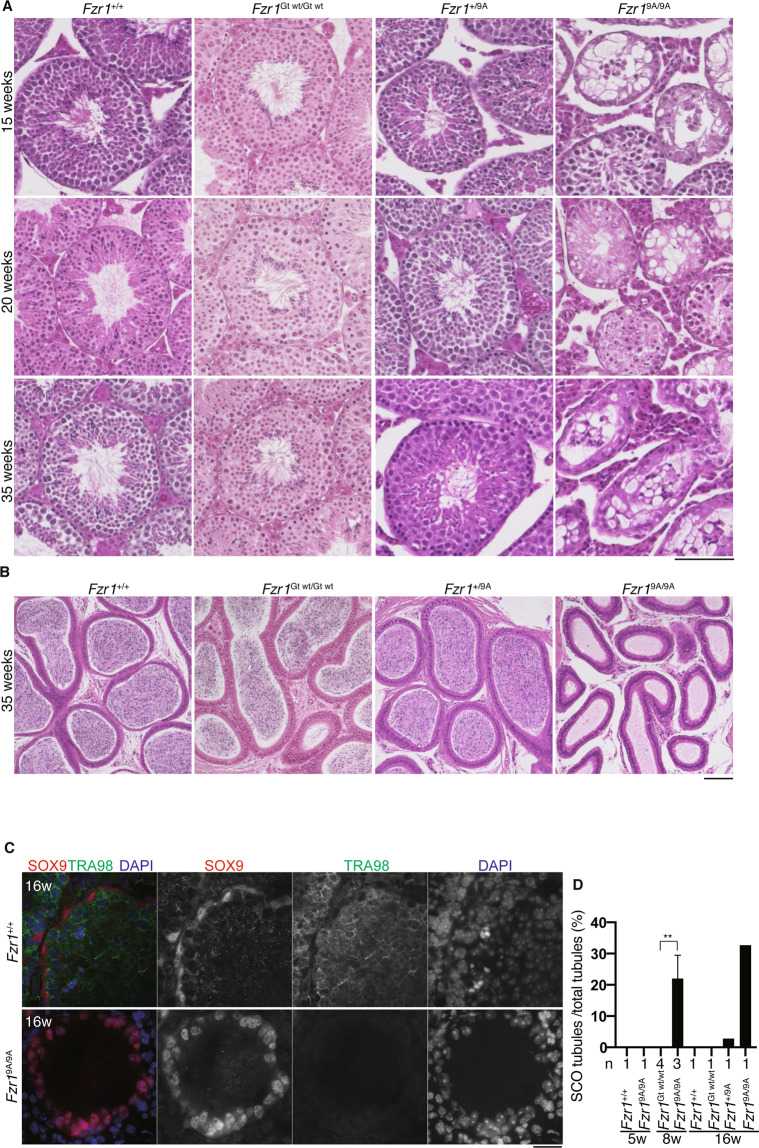
Sertoli cell-only phenotype appeared in aged *Fzr1*^9A/9A^ KI testes. **(A)** Hematoxylin and Eosin stained histological sections of seminiferous tubules in WT, *Fzr1*^Gt wt/Gt wt^, *Fzr1*^+/9A^ and *Fzr1*^9A/9A^ KI mice at the indicated ages. Scale bar: 100 μm. **(B)** Hematoxylin and Eosin stained sections of the epididymis in WT, *Fzr1*^Gt wt/Gt wt^, *Fzr1*^+/9A^ and *Fzr1*^9A/9A^ KI mice (35-week old). Scale bar: 100 μm. **(C)** Seminiferous tubule sections of indicated genotypes (5, 8 and 16-week-old) were stained for SOX9 (Sertoli cell marker), TRA98(germ cell marker) and DAPI. **(D)** Quantification of the seminiferous tubules of indicated genotypes (5, 8 and 16-week-old) that show Sertoli cell-only (SCO) phenotype per total tubules. n indicates the number of examined animals. **p < 0.01 (t-test). Scale bar: 25 μm.

We noticed that the loss of germ cells was more severe in aged *Fzr1*^9A/9A^ KI seminiferous tubules, with residual spermatogonia, spermatocytes and Sertoli cells remaining along the basement compartment of the tubules (Fig. 6A), which was accompanied by complete absence of spermatozoa in the epididymis (Fig. 6B). Immunostaining of the aged *Fzr1*^9A/9A^ KI seminiferous tubules revealed that TRA98 positive cells were lost while SOX9 positive cells remained in a subpopulation of the tubules, suggesting that germ cell populations were depleted (Fig. 6C). This trend was further augmented at older age in *Fzr1*^9A/9A^ KI seminiferous tubules (Fig. 6D). The aged *Fzr1*^9A/9A^ KI seminiferous tubules showed a severe loss of male germ cells, which was similar to Sertoli cell-only phenotype. This implied spermatogonia was depleted over a long period of time in *Fzr1*^9A/9A^ KI testis. Therefore, it is plausible that phosphorylation of FZR1 is required also for maintaining the spermatogonia population.

## Discussion

### Little impact of Fzr1^9A/9A^ KI allele on somatic cells

Contrary to previous observation in human cell culture study^16,17^, *Fzr1*^9A/9A^ KI mice showed no overt phenotype in mouse somatic tissues. This suggests that somatic cells undergo normal mitotic cell cycle in *Fzr1*^9A/9A^ KI mice, despite the constitutive activity of APC/C^FZR1^. It is possible that premature degradation of APC/C^FZR1^ specific substrates, such as cyclin A, cyclin B, DNA replication regulators (CDC6 and Geminin) and mitosis regulators (Aurora A, Aurora B, PLK1, CDC20 and SGO1) might be compensated by robust balance of cell cycle regulators in somatic tissues in the mutant mice.

It was demonstrated that while maternal store of FZR1 was not essential for completion of oocyte meiosis II following fertilization, loss of function of both maternal and paternal *Fzr1* alleles led to developmental defects in early embryo, where aberrant two independent spindles were formed following pronuclear fusion and the chromosomes failed to mix in 1-cell zygotes^33^. Thus, FZR1 is essential for development of early embryo. In contrast, since homozygous *Fzr1*^9A/9A^ KI animals were normally produced with expected mendelian ratio, it is plausible that maternal store of constitutive active APC/C ^FZR1–9A^ had little impact on oocyte meiosis or development of early embryo.

### Sexual dimorphisms of Fzr1^9A/9A^ KI phenotype

**Figure 7:**
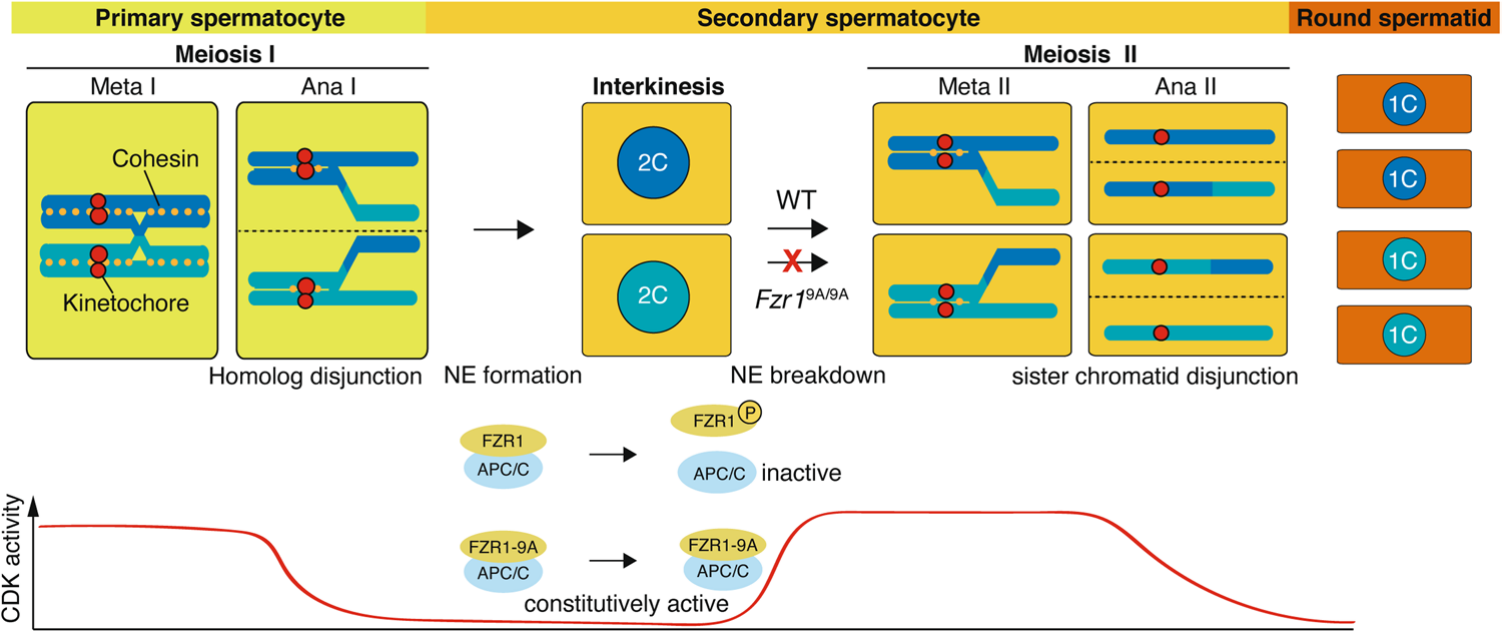
A schematic model of phosphorylation of FZR1 in meiosis II entry. (**A**) schematic shows progression of spermatocytes from meiosis I to meiosis II in male. CDK activity is elevated in metaphase I and metaphase II. Phosphorylation of FZR1 by CDK and its dissociation from APC/C are required for the entry into meiosis II. In *Fzr1*^9A/9A^ KI male, non-phosphorylatable FZR1 constitutively binds and activates APC/C. This results in unscheduled degradation of APC/C^FZR1^ substrates, that are required for entry into meiosis II. NE breakdown: nuclear envelope breakdown. 1 C and 2 C indicate DNA contents.

In male germ cells, it was shown that loss of function of FZR1 led to failure of spermatocytes to progress beyond zygotene and raised higher Cyclin B1 levels, indicating that APC/C ^FZR1^ activity is essential for progression of meiotic prophase^20^. Crucially, constitutively active APC/C ^FZR1–9A^ showed little impact on the progression of meiotic prophase (Fig. 3) but resulted in failure of spermatocytes to enter meiosis II, consequently producing 2 C DNA-content secondary spermatocyte-like cells (Fig. 7). This phenotype is reminiscent of the observation that large round spermatid-like cells were accumulated in *Meikin* KO testes, where meiosis II was blocked as a result of failure in reductional chromosome segregation during meiosis I^31^. The presence of PNA positive 2 C DNA-content cells (Figs. 4 and 5) suggested that the *Fzr1*^9A/9A^ KI spermatocytes were not simply arrested before meiosis II. At least, the *Fzr1*^9A/9A^ KI spermatocytes reached interkinesis but failed to enter meiosis II, which is similar to a phenomenon “mitosis skip” caused by premature activation of APC/C ^FZR1^^34^. FZR1 should be once released from APC/C by CDK-mediated phosphorylation after meiosis I to allow critical substrates accumulate for meiosis II entry. Otherwise, such substrates will be destroyed precociously by constitutively active APC/C ^FZR1^. It should be mentioned that despite failure in meiosis II entry, the *Fzr1*^9A/9A^ KI spermatocytes still showed an ability to undergo spermiogenesis. This implies that meiotic cell cycle program is genetically separable from spermiogenesis.

In female, loss of function of FZR1 led to impairment in progression of meiotic prophase, and consequently reduced the number of primordial follicle pool^20^. Furthermore, loss of function of FZR1 accelerated meiotic resumption of GV oocytes^21,22,27^, and elevated chromosome missegregation at meiosis I due to failure in removal of SGOL2 from the chromosome arms^24^. Maternal store of FZR1 is not essential for completion of oocyte meiosis II following fertilization^33^.

In female, non-phosphorylatable mutation of FZR1 did not exhibit apparently overt impact on female fertility, showing sexual dimorphism of the *Fzr1*^9A/9A^ allele. However, we still do not officially exclude the possibility that constitutively active APC/C ^FZR1–9A^ may give rise to more subtle effect on meiotic resumption of GV oocytes or chromosome missegregation at meiosis I, which will be a motive for future investigation using *in vitro* oocyte culture. The observed difference of *Fzr1*^9A/9A^ KI phenotypes in male and female might be attributed to the sexual difference in the mode of meiosis I-meiosis II transition. In male, spermatocytes pass through a transient interphase-like state^32^ with chromosome de-condensation and reassembly of nuclear membrane after completion of meiosis I. In female, chromosomes are persistently condensed without nuclear compartmentalization after completion of meiosis I until metaphase II. Since CDK-mediated phosphorylation of FZR1 inactivates APC/C ^FZR1^ through preventing its association with APC/C^10–12,16,17^, it is possible that APC/C ^FZR1^-mediated degradation of a critical regulator for male meiosis II entry is prevented by CDK-mediated phosphorylation, which requires further investigations. Alternatively, constitutive binding of FZR1–9A may compete with another activator CDC20 for the association with APC/C before meiosis II, which in turn raises a situation that APC/C ^CDC20^ specific substrates fail to undergo destruction in meiosis II.

### Impact of Fzr1^9A/9A^ KI allele on spermatogonial population in aged male

We have also shown that the *Fzr1*^9A/9A^ KI testes show Sertoli-cell only phenotype at older age (Fig. 6). Paradoxically, in the absence of APC/C ^FZR1^ activity, spermatogonial population was progressively lost at adulthood showing Sertoli-cell only phenotype^20^. Although the impact on APC/C ^FZR1^ activity should be opposite in *Fzr1* KO and *Fzr1*^9A/9A^ KI testes, the consequent phenotypes were apparently similar between those testes (Fig. 6). Because APC/C ^FZR1^ activity prevents mitotic cell cycle from premature S-phase onset and from spontaneously entering quiescence^35,36^, it is assumed that spermatogonia undergo precocious quiescence in *Fzr1* KO testis^20^. On the other hand, depletion of germ cells observed in adult *Fzr1*^9A/9A^ KI testes (Fig. 6) may imply that constitutive APC/C ^FZR1–9A^ activity enforces persistent proliferation of spermatogonia, prematurely exhausting spermatogonial population. Thus, CDK-mediated phosphorylation of FZR1 is required for sustaining spermatogonia over a long period of time.

Overall, temporal regulation of FZR1 by phosphorylation is essential for the maintenance of spermatogonia population and meiosis II entry in male germ cells.

## Materials and Methods

### Animal experiments

*Fzr1*^9A/9A^ KI and *Fzr1* ^*Gt* wt/ *Gt* wt^ KI mice were 129/C57BL6 mixed genetic background. Whenever possible, each knockin animal was compared to littermates or age-matched non-littermates from the same colony, unless otherwise described. Since *Fzr1*^+/+^, *Fzr1*^Gt wt/Gt wt^ KI, *Fzr1*^+/9A^ KI mice showed indistinguishable phenotype in fertility and testes morphology, they were used as controls when compared to *Fzr1*^9A/9A^ KI mice. All the animal methods were carried out in accordance with relevant guidelines and regulations. Animal experiments were approved by the Institutional Animal Ethics Committees of Keio University (approval 16037-1) and Kumamoto University (approval F28-078, A2020-006, A30-001, A28-026).

### Generation of *Fzr1* knock-in mouse and genotyping

*Fzr1*^+/GT^ allele was generated in TT2 embryonic stem (ES) cell line^37^ by integration of the pU-17 exchangeable GT vector^29^ into the *Fzr1* locus^30^. Characterization of the vector insertion site was performed by 5′ rapid amplification of cDNA ends (5′ RACE) and plasmid rescue experiments. Genotyping of the mutant mice was performed using a PCR protocol based on the primers Gs4 (5′ -CCTCCACTACAGCAGCACG-3′), Gas7 (5′-CTCCAAGGCCTTTGTGAGGC-3′), and SA6as (5′-CCGGCTAAAACTTGAGACCTTC-3′). For detection of the *Fzr1*–*β-geo* fusion mRNA, oligo(dT)-primed cDNAs derived from mutant mice were subjected to PCR using the primers 5NC-s (5′-TGTTCCTGGGACCGGCGGGAAC-3′) and LZUS-3 (5′-CGCATCGTAACCGTGCATCT-3′). The amplification product was cloned into the TA cloning vector and sequenced.

To produce ES cells in which the *β-geo* gene cassette of *Fzr1*^+/GT^ cells was replaced with cDNA encoding mouse wild type *Fzr1* (*Fzr1*^*Gt wt*^) *or Fzr1-9A* (*Fzr1*^*9A*^), we introduced the P17/FZR1 replacement vector together with pCAGGS-Cre^38^ into *Fzr1*^+/GT^ ES cells using electroporation. The P17/FZR1 replacement vector was designed to insert full length cDNA of mouse *Fzr1*^*Gt wt*^ *or Fzr1*^*9A*^ between 5′ *lox* 66 and 3′ *lox* P. Point mutations that substituted Thr/Ser codons with Ala codons were introduced into *Fzr1* cDNA (Eurofins), generating the P17/FZR1-9A replacement vector. After electroporation, ES cells were cultured in medium containing puromycin for 1 day to isolate cell lines that had undergone Cre-mediated recombination. Puromycin selection was performed twice at a 2-day interval. To detect the expression from the *Fzr1*^*wt*^ and *Fzr1*^*9A*^ knock-in (KI) alleles in the recombinant ES cell lines, we performed reverse transcription-PCR (RT-PCR) analysis using the primers 5NC-s2 (5′-TCGAACAGGCGCGGCGTGTT-3′) and mFzr as2 (5′-ATAGTCCTGGTCCATGGTG GAG-3′). The PCR product was cloned into the pGEM-T easy vector (Promega) and sequences were verified. Chimera mice were generated by morula aggregation (host ICR) of recombinant ES cells. Chimeric males were mated to C57BL/6N females and the progenies were genotyped by PCR using the following primers. Gs4 (5′-CCTCCACTACAGCAGCACG-3′) and Gas7 (5′-CTCCAAGGCCTTTGTGAGGC-3′) for the wild-type allele (0.4 kb).

Gs4 and SA6as (5 -CCGGCTAAAACTTGAGACCTTC-3′) for the knock-in allele (0.7 kb).

C57BL-mFzr1-9A GT (*Fzr1*^9A/9A^) and CB-Fzr1-wt GT (*Fzr1*^*Gt* wt/ *Gt* wt^) knockin mouse lines generated in this study have been deposited to Center for Animal Resources and Development (CARD) with ID3000 and ID1748, respectively.

### Preparation of testis extracts and immunoprecipitation

Testis extracts were prepared as described previously^39^. Briefly, testicular cells were suspended in low salt extraction buffer (20 mM Tris-HCl [pH 7.5], 100 mM KCl, 0.4 mM EDTA, 0.1% TritonX100, 10% glycerol, 1 mM β-mercaptoethanol) supplemented with Complete Protease Inhibitor (Roche). After homogenization, the soluble chromatin-unbound fraction was separated after centrifugation at 100,000 *g* for 30 min. The solubilized chromatin fraction was collected after centrifugation at 100,000 *g* for 30 min at 4 °C.

The endogenous APC/C was immunoprecipitated from chromatin-unbound fraction of *Fzr1*^+/+^, *Fzr1*^Gt wt/Gt wt^, *Fzr1*^9A/9A^ mice testes, using 2 µg of mouse anti-CDC27 antibody and 50 µl of protein A-Dynabeads (Thermo-Fisher). The beads were washed with low salt extraction buffer. The bead-bound proteins were eluted with 40 µl of elution buffer (100 mM Glycine-HCl [pH 2.5], 150 mM NaCl), and then neutralized with 4 µl of 1 M Tris-HCl [pH 8.0].The immunoprecipitated proteins were run on 4-12% NuPAGE (Thermo-Fisher) in MOPS-SDS buffer and immunoblotted. For the immunoblot of testes extracts, whole lysates were prepared in RIPA buffer and run on 8% Laemmli SDS-PAGE in Tris-Glycine-SDS buffer. Immunoblots were detected by secondary antibody VeriblotBlot for IP detection reagent (HRP) (ab131366, abcam, 1:3000 dilution), ECL rabbit IgG HRP-linked F(ab’)2 fragment, ECL mouse (NA9340, GE, 1:3000 dilution) or IgG HRP-linked F(ab’)2 fragment (NA9310, GE, 1:3000 dilution). Immunoblot image was developed using ECL prime (GE healthcare) and captured by FUSION Solo (VILBER).

### Antibodies

The following antibodies were used for immunoblot (IB) and immunofluorescence (IF) studies: rabbit anti-Actin (IB, 1:1000, Sigma A2066), mouse anti-CDC27 (IB, 1:1000, Abcam: ab10538), rabbit anti-FZR1 (IB, IF, 1:1000, Abcam: ab118939), rabbit anti-CDC20 (IB, 1:1000, Bethyl:A301-180A), rabbit anti-SOX9 (IF, 1:1000, Milipore: Ab5535), rabbit anti-CyclinB1(IB, 1:1000, Santa Cruz: sc-595), mouse anti-PLK1(IB, 1:1000, Abcam ab17056), rabbit anti-SYCP1 (IF, 1:1000, Abcam ab15090), rabbit anti-γH2AX (IF, 1:1000, Abcam ab2893), rat anti-TRA98 (IF, 1:1000, ab82527), rabbit anti-CENPC^39^, rabbit anti-MEIKIN (IF, 1:1000)^31^, rat anti-SYCP3^40^, guinea pig anti-H1t (IF, 1:2000, kindly provided by Marry Ann Handel).

### Histological analysis

For hematoxylin and eosin staining, testes, epididymis and ovaries were fixed in 10% formalin or Bouin solution, and embedded in paraffin. Sections were prepared on CREST-coated slides (Matsunami) at 6 μm thickness. The slides were dehydrated and stained with hematoxylin and eosin.

For Immunofluorescence staining, testes were embedded in Tissue-Tek O.C.T. compound (Sakura Finetek) and frozen. Cryosections were prepared on the CREST-coated slides (Matsunami) at 8 μm thickness, and then air-dried and fixed in 4% paraformaldehyde in PBS at pH 7.4. The serial sections of frozen testes were fixed in 4% PFA for 5 min at room temperature and permeabilized in 0.1% TritonX100 in PBS for 10 min. The sections were blocked in 3% BSA/PBS, and incubated at room temperature with the primary antibodies in a blocking solution. After three washes in PBS, the sections were incubated for 1 h at room temperature with Alexa-dye-conjugated secondary antibodies (1:1000; Invitrogen) in a blocking solution. For paraffin embedded section, sections were deparaffinized, rinsed in distilled water and incubated with PBS for 30 minutes at 37°C. Then, sections were incubated in PBS containing for Proteinase K for 30 minutes at 37°C and rinsed in distilled water. After treatement, immunofluorescence staining were performed with same procedure of frozen tissue sections.

TUNEL assay was performed using MEBSTAIN Apoptosis TUNEL Kit Direct (MBL 8445). DNA was counterstained with Vectashield mounting medium containing DAPI (Vector Laboratory). Lectin staining was done using Lectin from *Arachis hypogaea* FITC Conjugate (IF, 1:1000, Sigma: L7381).

### Immunostaining of spermatocytes

Spread nuclei from spermatocytes were prepared as described^41^. Briefly testicular cells were suspended in PBS, then dropped onto a slide glass together with an equal volume of 2% PFA, 0.2% (v/v) Triton X-100 in PBS, and incubated at room temperature in humidified chamber. The sides were then air-dried and washed with PBS containing 0.1% Triton-X100 or frozen for longer storage at −80 °C. The serial sections of frozen testes were fixed in 4% PFA for 5 min at room temperature and permeabilized in 0.1% TritonX100 in PBS for 10 min. The slides were blocked in 3% BSA/PBS, and incubated at room temperature with the primary antibodies in a blocking solution. After three washes in PBS, the sections were incubated for 1 h at room temperature with Alexa-dye-conjugated secondary antibodies (1:1000; Invitrogen) in a blocking solution. DNA was counterstained with Vectashield mounting medium containing DAPI (Vector Laboratory).

### Fluorescence *in situ* hybridization (FISH) on immunostained nuclei

For immuno-FISH, structurally preserved nuclei (SPN) from spermatocytes were prepared as described^41^ with modification. Briefly testicular cells were collected in PBS by mincing seminiferous tubules into small pieces with fine-tipped tweezers and then pipetting. After removal of tissue pieces, the cell suspension was filtered through a Cell strainer (BD Falcon) to remove debris. The cell suspension (~5 μl) was dropped onto a MAS-coated slide glass (Matsunami) and fixed with 10 μl of 2% Paraformaldehyde (PFA)/100 mM sucrose in PBS for 10 min followed by the addition of 1.5 μl of 1.25 M Glycine/PBS, and then air-dried at room temperature. Immediately before they were completely air-dried, the slide glasses were washed with PBS containing 0.1% Triton-X100 or frozen for longer storage at −80 °C.

Immuno-stained samples of SPN were fixed in 4% paraformaldehyde for 5 min, washed with PBS, and subjected to sequential dehydration through 70%, 80%, 90%, 100% ethanol. Immuno-stained SPNs were denatured in 50% formamide, 2x SSC at 72 °C for 10 min. Hybridization was conducted with a fluorescence-labeled point probe in buffer containing 50% formamide, 2x SSC, 20% dextran sulfate at 37 °C for 12-16 h. The slides were washed sequentially at room temperature in 2x SSC for 1 min, 0.4x SSC/0.3% Tween20 solution for 2 min, and 2xSSC at room temperature for 1 min. The mouse point probe derived from BAC clone RP23-6I6 detects the mid region of chromosome 3. A biotinylated point probe that detects X chromosome and a FITC-labeled painting probe that detects whole Y chromosome (MXY-10, Chromosome Science Labo, Hokkaido, Japan) were used. The biotinylated X chromosome point probe was detected by Alexa555-streptavidine (Thermo, S21381).

### Imaging

Immunostaining images were captured with DeltaVision (GE Healthcare). The projection of the images was processed with the SoftWorx software program (GE Healthcare). For Fig. S2B, images were captured with Zeiss LSM-710 confocal microscope and processed with ZEN software. All images shown were Z-stacked. Bright field images and immunofluorescent images for counting seminiferous tubules, were captured with BIOREVO BZ-X710 (KEYENCE), and processed with BZ-H3A program.

All methods were carried out in accordance with relevant guidelines and regulations.

## Supplementary information

Supplementary Information.
