# Supplementary material for: Phosphorylation of the Anaphase Promoting Complex activator FZR1/CDH1 is required for Meiosis II entry in mouse male germ cell
Source: Sci Rep. 2020 Jun 22;10:10094. doi: 10.1038/s41598-020-67116-0 (PMC7308413; doi:10.1038/s41598-020-67116-0)
Supplement: Supplementary file 1 — Supplementary Information. [file 41598_2020_67116_MOESM1_ESM.pdf]

Supplementary Figures

Phosphorylation of the Anaphase Promoting Complex activator FZR1/CDH1 is required for Meiosis II entry in mouse male germ cell

Nobuhiro Tanno[1 2], Shinji Kuninaka[2], Sayoko Fujimura[3], Kazumasa Takemoto[1], Kaho Okamura[1], Naoki Takeda[4], Kimi Araki[4 5], Masatake Araki[4], Hideyuki Saya[2] and Kei-ichiro Ishiguro[1]*

1 Department of Chromosome Biology, Institute of Molecular Embryology and Genetics (IMEG), Kumamoto University, Kumamoto, 860-0811 Japan
2 Division of Gene Regulation, Institute for Advanced Medical Research, Keio University School of Medicine, 160-8582, Japan
3 Liaison Laboratory Research Promotion Center, IMEG, Kumamoto University
4 Institute of Resource Development and Analysis, Kumamoto University, Kumamoto, 860-0811 Japan
5 Center for Metabolic Regulation of Healthy Aging, Kumamoto University, Kumamoto, 860-0811 Japan
* correspondence : ishiguro@kumamoto-u.ac.jp

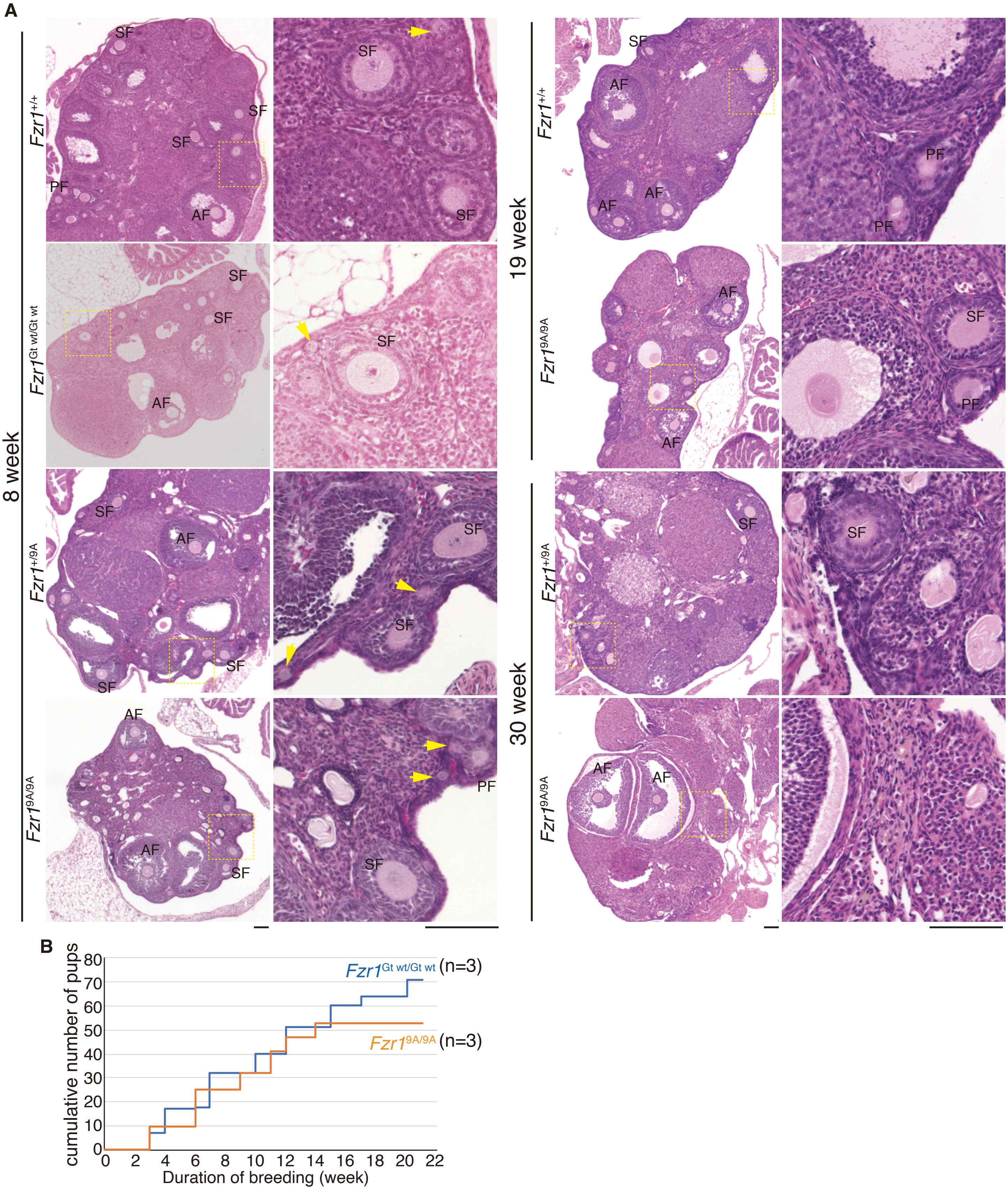

Supplementary Figure 1. ***Fzr1***$^{9A/9A}$ **knockin females showed no overt defects in adult ovaries**

(A) Hematoxylin and Eosin stained sections of WT, *Fzr1*$^{Gt\ wt/Gt\ wt}$, *Fzr1*$^{+/9A}$ and *Fzr1*$^{9A/9A}$ KI ovaries at the indicated age. Enraged images of the area indicated by yellow dot line are shown on the right. arrow heads: primordial follicle. PF: primary follicle, SF: secondary follicle, AF: antral follicle. Scale bars: 100µm

(B) Cumulative number of pups born from *Fzr1*$^{Gt\ wt/Gt\ wt}$ KI (n=3, all 6-week old at the start point of mating) and *Fzr1*$^{9A/9A}$ KI (n=3, 9-week, 11-week, 11-week old at the start point of mating) females. Starting point of mating was set to 0 week.

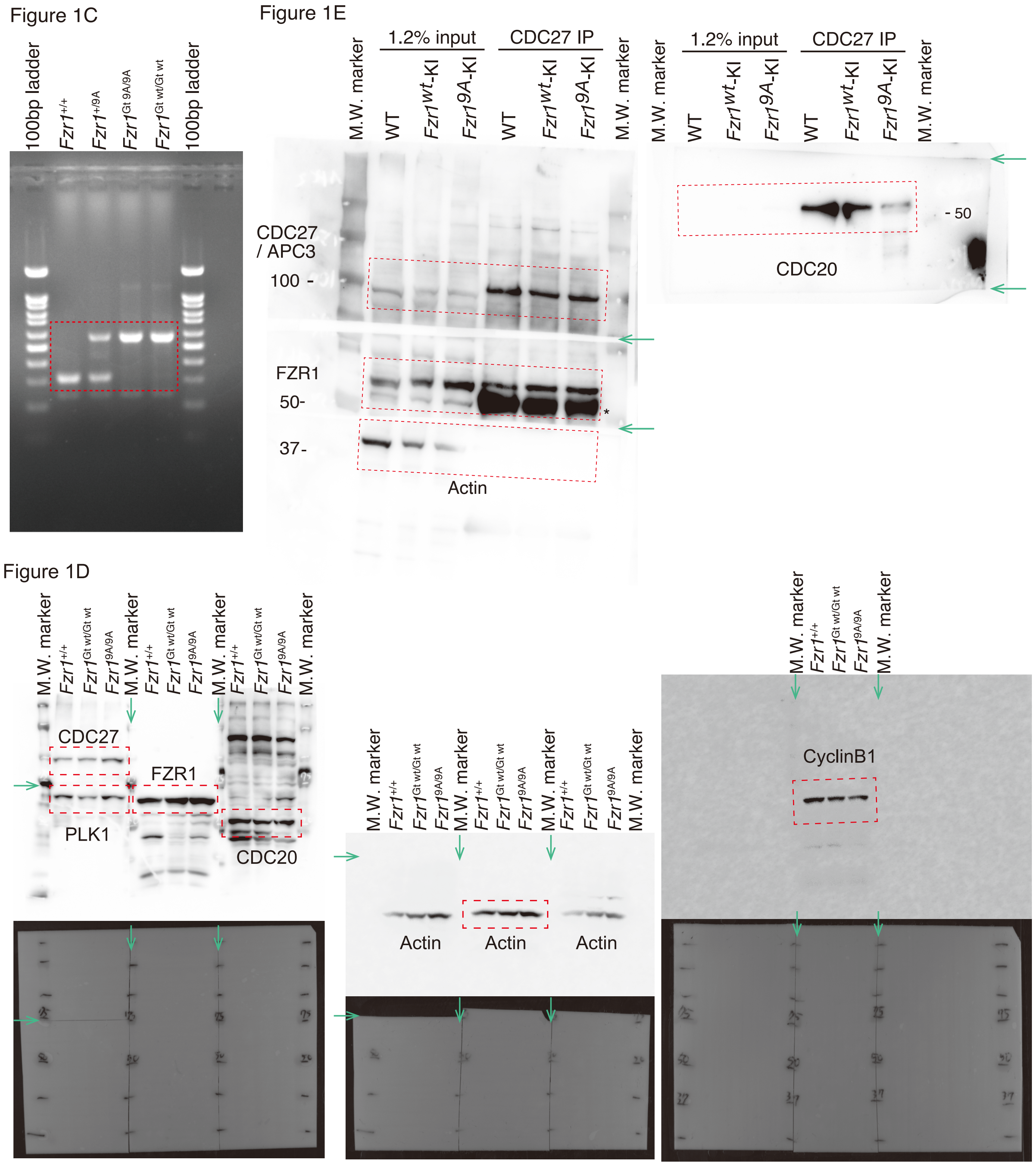

Supplementary Figure 2. Full-length / uncropped images

Full-length / uncropped images of agarose gel (Fig1C) and immunoblots (Fig1D, E) are shown. For immunoblot of Fig1D, the same samples of testis extracts were run on the same gel in triplicate and blotted to the same membrane. Blotted membrane was cut into four parts (indicated by green arrows) so that different proteins (CDC27, PLK1, FZR1, CDC20) could be simultaneously probed with different antibodies. Each piece of membrane was stripped and reprobed with Actin antibody. For detection of CyclinB1, the same samples of testis extracts were run on another gel, blotted and probed with CyclinB1 antibody. The same membrane was stripped, cut according to molecular weight marker and reprobed with Actin antibody. The images of immunoblots taken under chemiluminescence (upper) and the corresponding membranes taken under EPI white light (lower) are shown.

For immunoblot of Fig1E, input extracts and CDC27-IPs were run on the same gel and blotted. Blotted membrane was cut into three parts according to molecular weight markers (indicated by green arrows) so that different proteins (CDC27, FZR1, ACTIN) on the same lanes could be simultaneously probed by different antibodies. CDC20 was separately probed on another membrane on which the same samples of input and CDC27-IPs were blotted.
